# Recent advances in physiological priming of spermatozoa

**DOI:** 10.4103/0974-1208.74167

**Published:** 2010

**Authors:** Satendra Singh

**Affiliations:** University College of Medical Sciences, Delhi, India

Sir,

The molecular mechanisms implicated in the initiation of capacitation are poorly understood, and the review by Sheriff and Ali made an interesting read.[1] However, the review quoted only one citation after 2002 and as such few promising articles were inadvertently missed.

It would have been interesting to include Travis and Kopf’s lipid-poor model for cholesterol efflux.[2] Recent investigators have put forward an expansion of Travis and Kopf’s lipid exchange model for the delivery of Glycosyl phosphatidylinositol (GPI)-linked proteins to sperm membranes through clusterin (CLU).[3] CLU has been implicated in lipid efflux from the sperm plasma membrane during capacitation. Its role in removal and delivery of SPAM1 has physiological relevance and could lead to successful fertilization by the addition of relevant GPI-linked proteins. A further breakthrough was identifying the missing link between protein kinase A phosphorylation and cholesterol efflux as apolipoprotein A-I binding protein.[4]

Proteomic analysis on detergent-resistant membranes of sperm cells indicated that capacitation induces a lipid raft concentration,[5] rather than a disintegration of lipid rafts as earlier thought. The precise signaling mechanism has remained elusive, but recent reviews have elucidated possible mechanisms concerned in regulating sperm capacitation and the acrosome reaction.[6,7]

Within the female genital tract, spermatozoa interact with four glycoforms before fertilizing the oocyte.[8] Understanding of these recently found glycodelins may facilitate development of novel strategies for fertility regulation [Table 1].

**Table 1:** Glycodelin isoforms affecting capacitation

Glycoform	Source	Function	Effect
Glycodelin-S	Seminal plasma	Suppresses albumininduced cholesterol efflux from the spermatozoa	Regulates the initiation of capacitation
Glycodelin-A	Oviductal fluid	Suppresses extracellular signalregulated kinase. Inhibit spermatozoazona pellucida binding by interacting with sperm surface fucosyltransferase-5	Making the spermatozoa more sensitive to zona pellucidainduced acrosome reaction. Physiological implication unknown
Glycodelin-F	Follicular fluid	Suppresses progesterone-induced acrosome reaction. Inhibit spermatozoazona pellucida binding by interacting with sperm surface fucosyltransferase-5	Prevents premature acrosome reaction. Physiological implication unknown
Glycodelin-C	Cumulus matrix	Removes inhibitory activities of glycodelin-A and glycodelin-F on spermatozoa-zona pellucida binding	Enhances spermatozoa-zona pellucida binding

Finally, olfaction plays a critical role in sperm chemotaxis. The first evidence in favor of this hypothesis was provided by the identification of hOR17-4, a testicular olfactory receptor mediating human sperm chemotaxis in various bioassays.[9] Subsequent finding of mOR23 in mouse sperm confirmed olfactory-like signaling mechanisms in mammalian sperm.[10] This odorant-receptor-mediated signaling ‘could set the stage for pioneering future applications in procreation and/or contraception.’[9]
